# Knowledge, attitude, and practice of Iranian adults toward Persian Medicine: a national survey

**DOI:** 10.1186/s12906-024-04461-x

**Published:** 2024-05-21

**Authors:** Alireza Abbassian, Mohammad Hossein Abbaassi, Zahra Pouraskari, Farshid Alaedini, Abbas Abbasi-Ghahramanloo, Elham Emaratkar, Mohammad Hossein Ayati, Ebrahim Khadem, Meysam Shirzad, Malihe Tabarrai, Rasool Choopani, Mojgan Tansaz, Jila Sadighi, Hossein Rezaeizadeh, Shahin Akhondzadeh, Mahmood Khodadoost

**Affiliations:** 1 Department of Traditional Medicine, School of Persian Medicine, Tehran University of Medical Sciences, Tehran, Iran; 2 Department of Community Oral Health, School of Dentistry, Tehran University of Medical Sciences, Tehran, Iran; 3 Private researcher, Tehran, Iran; 4 Department of Public Health, School of Health, Ardabil University of Medical Sciences, Ardabil, Iran; 5 Department of Persian Medicine, Faculty of Medicine, Shahed University, Tehran, Iran; 6 Department of History of Medicine, School of Persian Medicine, Tehran University of Medical Sciences, Tehran, Iran; 7 Guest Professor, Beijing University of Chinese Medicine, Beijing, China; 8 Department of Persian Medicine, School of Persian Medicine, Shahid Beheshti University of Medical Sciences, Tehran, Iran; 9 Family Health Research Group, Health Metrics Research Center, Institute for Health Sciences Research, ACECR, Tehran, Iran; 10 Psychiatric Research Center, Roozbeh Hospital, Tehran University of Medical Sciences, Tehran, Iran

**Keywords:** Knowledge, Attitude, Practice, Persian medicine, Complementary therapies, Traditional medicine

## Abstract

**Background:**

Previous researches conducted in both developed and developing countries have demonstrated a rising trend in the utilization of complementary and alternative medicine. The World Health Organization has underscored the importance of studying the prevalence and determinants of such alternative practices. This study delves into the knowledge, attitudes, and practices of Iranian adults towards Persian medicine, a distinct form of complementary and alternative medicine, through a national survey for the first time.

**Methods:**

A total of 2882 Iranian adults (aged ≥ 15 years) were randomly chosen from all regions. Data were gathered through structured door-to-door interviews using a survey questionnaire, wherein cases were selected randomly based on postal codes, and interviewers completed the forms at the participants’ residences.

**Results:**

Approximately 46% of the subjects exhibited familiarity with Persian Medicine. The study revealed that the primary sources of knowledge about Persian Medicine were family, relatives, and friends, with only 2.9% indicating medical staff as their source of information. Furthermore, the study indicated that 21% of participants expressed a strong interest in using Persian Medicine, while 30.3% did not. When comparing Persian medicine to modern medicine, respondents indicated that Persian medicine is more cost-effective and has fewer side effects, yet modern medicine is more widely used globally; although, the majority responded “I don’t know” to other questions. The results also demonstrated that approximately 37% of the participants had a history of Persian Medicine utilization in their lifetime.

**Conclusion:**

This study revealed that Iranian adults have low reliable knowledge (from medical staff, Persian medicine books and other publications) and limited familiarity with Persian medicine, with about one third of the participants expressing disinterest (attitude) and over half of them having not utilized this form of medicine (practice).

## Introduction

Complementary and alternative medicine (CAM) encompasses diverse medical approaches beyond conventional medicine. Complementary medicine refers to non-mainstream practices used alongside conventional medicine, while alternative medicine refers to non-mainstream practices used in place of conventional medicine [1]. The World Health Organization (WHO) underscores the importance of studying the prevalence and determinants of CAM use [2]. Previous studies have indicated that CAM utilization ranges from 9.8 to 76% in high-income countries [3]. The escalating incidence of chronic diseases has led to increased CAM use in recent years [4]. Prior researches have shown a growing trend in the adoption of complementary medicine in both developed and developing countries [5–7].

It is estimated that approximately 33% of people use traditional medicine products to address common issues such as back pain, anxiety, and depression [7]. Roughly 40% of the general population in the United States of America (USA) engages with some form of alternative medicine [8]. Findings from a telephone survey in the United Kingdom (UK) regarding CAM usage showed a one-year prevalence of 20% [9]. Data indicates that the majority of CAM usage is in many developing countries. For instance, in Colombia, Chile, and various African countries, 42%, 48%, and over 80% of the population reported using CAM [10].

In Iran, there exists a rich traditional medicine known as Persian Medicine (PM) with a history of more than 3000 years for treating medical conditions [11]. In recent centuries, traditional medicine in Iran encountered legal challenges. However, it never completely disappeared from the lives of the people and remained an integral part of the culture, despite significant pressure from modern medicine [12]. Presently, numerous medications and procedures of PM are being researched [13–17]. However, there is no accurate estimation of traditional and PM utilization in Iran. For this reason, the current study aims to evaluate Iranian adults’ knowledge, attitudes, and practices concerning PM as a type of CAM in the first step.

## Methods

### Study Design & Sampling

This cross-sectional study was carried out in Iran in 2016, using a representative sample of adults aged 15 years or older. Based on the Ministry of Health classification, Iran is divided into 5 regions. Tehran city encompasses approximately 20% of Iran’s population itself, thus, we considered 3 regions from central region which Tehran city is located in it. Hence, we aimed to cover 7 regions for sampling. For determining the sample size, we considered a 95% confidence interval and 5% absolute error. Utilizing Cochrane’s formula, the sample size for each region was calculated as 384 persons, resulting in a total of 2688 participants nationwide. Additionally, we allowed for an 8% dropout rate based on our pilot study. Eventually, a total of 2902 participants were selected for the study. Participants from each region were chosen through simple random sampling based on household postal codes.

Data collection included structured door-to-door interviews. In door-to-door interviews, investigators went selected postal code and completed the questionnaire with a household’s member who was 15 years or older. We utilized a questionnaire composed of 68 questions in 4 main sections: (1) demographic characteristics (2), knowledge of PM (3), attitude toward PM, and (4) practice regarding PM.

The primary questionnaire was developed by 10 faculty members specializing in PM. This questionnaire underwent review in three expert panels to establish and confirm its validity. Furthermore, the survey questionnaire was tested by 30 individuals similar to the study participants, and its reliability was confirmed through a test-retest method. During the pilot study, input data was employed to refine the questionnaire, and questions that most participants found challenging to comprehend or respond to were removed.

Ten interviewers, each holding at least a Bachelor of Science in the medical fields, conducted the interviews after receiving 5 h of training from the research team and passing an exam in role-playing.

### Variables

The study’s outcomes focused on knowledge, attitude, and practice toward Persian Medicine. Demographic variables included gender, marital status, nationality, residency, and age.

### Ethical approval

At the time of study design and approval, there was no requirement to obtain an ethics approval ID for such studies, according to the regulations of the Ethics Committee of Tehran University of Medical Sciences. To ensure the validity of participant responses, strict confidentiality was assured, and participants’ anonymity was preserved through the use of anonymous questionnaires. Participants were also made aware of the voluntary nature of their participation and their right to refuse or skip any questions. Consequently, there were minimal instances of missing data noted in the tables, and 20 participants refused to participate in the study.

### Statistical analysis

We depicted qualitative and quantitative variables using frequency (percent) and mean ± standard deviation (SD). Single-factor and multi-factor analysis of variance were employed to ascertain the factors associated with participants’ knowledge, attitude, and practice. Significant variables identified in the single-factor model were included in the multi-factor model. The Chi-Square test was used to examine differences between groups. All analyses were conducted using SPSS-16 for Windows [SPSS, Chicago, IL, USA] and a *p*-value of ≤ 0.05 was deemed statistically significant.

## Results

In total, 2882 participants responded to the questions, with a mean age of 41.24 (SD = 15.90). The majority of respondents were female (56.0%), married (75.4%), of Iranian nationality (98.9%), urban residents (79.8%), and aged 31–45 years old (34.4%). Additional demographic characteristics are presented in Table 1.

**Table 1:** Demographic characteristics of participants

Characteristics	Number	%
**Gender**		
* Male*	1266	44
* Female*	1616	56
**Marital status**		
* Married*	2180	75.5
* Single*	533	18.7
* Other*	169	5.8
**Nationality**		
* Iranian*	2851	98.9
* Other*	31	1.1
***Residency***		
* Urban*	2297	79.8
* Rural*	585	20.2
***Age***		
* 30 and less*	862	29.9
* 31–45*	991	34.4
* 46–60*	641	22.2
* 60 and more*	388	13.5

Close to 46% (*N* = 1333) of participants were familiar with Persian Medicine (PM), with the most common source of their knowledge being family, relatives, and friends. Only 2.9% reported that medical staff were their source of knowledge. Table 2 presents the distribution of participants’ knowledge about PM.

**Table 2:** Distribution of knowledge of the participants toward PM in a sample of the Iranian population

Characteristics	Number	%
**Familiarity with PM**		
High*	58	2.0
* Medium*	473	16.4
* Low*	802	27.8
* No familiar*	1549	53.7
* Total*	2882	100
**Sources of knowledge about PM**		
* Media*	341	25.6
* books and other publications*	204	15.3
* Internet*	124	9.3
* Family, relatives, and friends*	610	45.8
* Medical staffs*	39	2.9
* Others*	15	1.1
* Total*	1333	100
**Do you know what PM includes?**		
* Yes*	694	52
* No*	384	28.8
* Miss*	255	19.1
* Total*	1333	100
**Can PM have side effects?**		
* Yes*	212	15.9
* no*	450	33.8
* Don’t know*	510	38.3
* Miss*	161	12
* Total*	1333	100

* *A high mean indicates a preference for using as the primary choice of treatment, while a medium mean suggests using it as complementary medicine alongside modern practices. A low mean indicates infrequent usage, and ‘no interest’ signifies no intention to use*

The knowledge score regarding PM was associated with gender, residency, and age. Table 3 illustrates the association between demographic characteristics and familiarity with PM.

**Table 3:** Demographic characteristics and Familiarity with Persian Medicine in a sample of the Iranian population

Characteristics	Familiarity with Persian Medicine				*P*-value	N
	High*	Medium	Low	No familiar		
	N(%)	N(%)	N(%)	N(%)		
**Gender**						
Male	28(2.2)	174(13.7)	310(24.4)	756(59.6)	*P* < 0.001	1268
Female	30(1.9)	299(18.5)	492(30.5)	793(49.1)		1614
**Residency**						
Rural	9(1.5)	66(11.3)	173(29.6)	336(57.5)	*P* = 0.002	584
Urban	49(2.1)	407(17.7)	329(27.4)	1213(52.8)		2298
**Age Groups**						
≤ 30	18(2.1)	160(18.6)	246(28.6)	436(50.7)	*P* < 0.001	860
31–45	27(2.7)	181(18.3)	274(27.7)	507(51.3)		989
46–60	8(1.2)	92(14.3)	191(29.8)	351(54.7)		642
> 60	5(1.3)	40(10.2)	91(23.3)	255(65.2)		391

* *A high mean indicates a strong inclination to use Persian Medicine as the primary treatment choice, while a medium mean suggests familiarity with using it as a complementary medicine alongside modern medical practices. A low mean reflects minimal familiarity or unwillingness to use Persian Medicine*

Table 4 indicates participants’ general attitudes toward PM.

**Table 4:** Distribution of attitude of the participants toward PM in a sample of the Iranian population

Characteristics	Number	%
**How much interested to use PM?**		
* High*	605	21.0
* Medium*	790	27.4
* Low*	613	21.3
* No interested*	874	30.3
* Total*	2882	100
**In what cases can use Persian medicine?**		
* For all disease instead common medicine*	176	6.1
* For some disease instead common medicine*	758	26.3
* For all disease aside common medicine*	277	9.6
* For some disease aside common medicine*	527	18.3
* For any disease instead common medicine*	35	1.2
* For any disease aside common medicine*	23	0.8
* Don’t know*	1052	36.5
* Miss*	37	1.3
* Total*	2882	100
**If you want use the PM what is the reason**		
* Both treatment and increase health overall*	709	24.6
* Faster effect*	297	10.3
* More effect*	308	10.7
* Doing some treatments by myself*	775	26.9
* In PM more time puts on me*	115	4
* Interest in experience new methods of treatment*	444	15.4
* Other*	95	3.3
* Miss*	135	4.7
* Total*	2882	100

The demographic characteristics and the tendency to use PM among the total sample are shown in Table 5. According to this table, there is a statistical association between residency and age groups with this tendency.

**Table 5:** Demographic characteristics and tendency to use PM in a sample of the Iranian population

Characteristics	How much tend to use CAM?				*P*-value	N
	High*	Medium	Low	No interested		
	N(%)	N(%)	N(%)	N(%)		
**Gender**						
Male	276(21.8)	343(27.1)	260(20.5)	389(30.7)	*P* = 0.689	1268
Female	329(20.4)	447(27.7)	353(21.9)	485(30.0)		1614
**Residency**						
Rural	119(20.4)	129(22.1)	137(23.5)	199(34.1)	*P* = 0.005	584
Urban	486(21.1)	661(28.8)	476(20.7)	675(29.4)		2298
**Age Groups**						
≤ 30	162(18.8)	258(30.0)	197(22.9)	243(28.3)	*P* < 0.001	860
31–45	224(22.6)	301(30.4)	199(20.1)	265(26.8)		989
46–60	141(22.0)	168(26.2)	151(23.5)	182(28.3)		642
> 60	78(19.9)	63(16.1)	66(16.9)	184(47.1)		391

* A high mean indicates a tendency to use as the primary choice of treatment, while a medium mean suggests a tendency to use it as complementary medicine alongside modern practices. A low mean implies a rare tendency to use, and ‘no interest’ signifies no inclination to use.

Table 6 showcases the participants’ attitudes regarding the comparison of traditional medicine and modern medicine. They stated that traditional medicine has a lower cost and fewer side effects, and modern medicine is more widely used in the world. However, the majority of responses to other questions were “I don’t know.”

**Table 6:** Persian medicine and modern medicine comparisons based on participants’ attitudes

Characteristics	Type of Medicine				
	Modern medicine	Persian Medicine	Non	don’t know	Miss
	N(%)	N(%)	N(%)	N(%)	N(%)
Lower cost	353(12.2)	1217(42.2)	126(4.4)	1129(39.2)	57(2.0)
fewer side effects	263(9.1)	1385(48.1)	58(2.0)	1131(39.2)	45(1.6)
More accessible	875(30.4)	863(29.9)	58(2.0)	1031(35.8)	55(1.9)
More hygienic	1095(38.0)	463(16.1)	51(1.8)	1188(41.2)	85(2.9)
More usage in world	1296(45.0)	220(7.6)	47(1.6)	1251(43.4)	68(2.4)
More ethical	525(18.2)	656(22.8)	57(2.0)	1559(54.1)	85(2.9)
Faster response to treatment	1000(34.7)	445(15.4)	65(2.3)	1310(45.5)	62(2.2)

The practice of PM is illustrated in Table 7. We asked about PM use. The results showed that about 36.9% of the participants have a history of using PM in their lifetime. The next question was a reflection of whether their practitioner had inquired about PM use. If PM users had not informed their practitioner, the reason for this was explored. The final question in this section related to the participants’ first choice when they are sick.

**Table 7:** Distribution of participants’ practice toward PM in a sample of the Iranian population

Characteristics	Number	%
**PM use(ever)**		
* Yes*	1064	36.9
* no*	1818	63.1
* Total*	2882	100
**Reflection of PM usage to physicians**		
* Yes*	375	35.2
* no*	596	56
* I don’t remember*	83	7.8
* Miss*	10	0.9
* Total*	1064	100^**^
**What is the reason for not informing PM usage to physicians**		
* My doctor does not believe in these methods*	55	8.1
* I didn’t think it was necessary*	425	62.4
* My doctor not paying attention to my words*	43	6.3
* Other*	22	3.2
* Miss*	136	20
* Total*	681	100
**What is the first thing you do when you are sick**		
* Visit a general practitioner*	1686	58.5
* Refer to the PM clinic*	159	5.5
* Using home remedies*	876	30.4
* Other*	95	3.3
* Miss*	66	2.3
* Total*	2882	100

Table 8 describes the relationship between demographic characteristics and PM use among participants. According to this table, PM utilization was significantly associated with gender and age groups.

**Table 8:** Demographic characteristics and use of PM in a sample of the Iranian population

Characteristics	Ever CAM use		*P*-value	N
	yes	no		
	N(%)	N(%)		
**Gender**				
Male	432(34.1)	836(65.9)	*P* = 0.005	1268
Female	632(39.2)	982(60.8)		1614
**Residency**				
Rural	215(36.8)	369(63.2)	*P* = 0.954	584
Urban	849(36.9)	1449(63.1)		2298
**Age Groups**				
≤ 30	281(32.7)	579(67.3)	*P* < 0.001	860
31–45	407(41.2)	582(58.8)		989
46–60	249(38.8)	393(61.2)		642
> 60	127(32.5)	264(67.5)		391

## Discussion

The current study assessed the knowledge, attitudes, and practices of Iranian adults towards Persian Medicine (PM). Our findings revealed that less than half of Iranian adults were familiar with PM, with only 2% demonstrating high knowledge of it. This figure contrasts with previous studies, which reported a 45 to 80% range of good knowledge about traditional medicine in other countries. Therefore, we can conclude that the adult population in Iran lacks adequate knowledge toward PM.

We discovered that the primary sources of knowledge about PM were family, relatives, and friends, while only 2.9% cited medical staff as their source of information. Comparatively, Molavi Vardanjani et al. found that 44.43% of pregnant women in Shiraz (south of Iran) were recommended to use complementary and alternative medicine (CAM) by relevant individuals [18]. Moeini et al. found that 63% of the population in Babol (located in northern Iran) chose complementary and alternative medicine (CAM) based on recommendations from family and relatives [19]. Al Akeel et al. mentioned that friends and the web played a significant role, with 48% and 23.5% respectively, as sources of knowledge for CAM in Saudi Arabia, while only 6% of participants obtained CAM information from practitioners [20].

Our study highlighted that approximately 18% of participants who were familiar with PM believed it could have potential side effects. For instance, Wassie et al. reported that about 11% of Merawi residents believed that traditional medicine had adverse effects [21]. This implies that despite a level of knowledge about PM, there is some skepticism toward this form of medicine among the Iranian population.

Furthermore, our findings revealed that Iranian adults believed traditional medicine to be more cost-effective and to have fewer side effects compared to modern medicine. On the other hand, they viewed modern medicine as more hygienic, having greater global usage, and providing quicker response to treatment compared to traditional medicine. Similarly. Moeini et al. elucidated that the primary reasons for utilizing complementary and alternative medicine (CAM) in the northern Iranian city of Babol are the perceived lower complication rates and the effectiveness of these methods [19]. Wassie et al. found that 26% of participants believed traditional medicines were more effective and safer than modern health services [21]. Singh et al. showed that 23.4% of the Indian population in South Africa perceived CAM as natural as and safer than medical care, with 15.6% choosing CAM due to the undesired adverse effects of modern medicine [22].

Our study also demonstrated that 37% of participants had a history of using PM in their lifetime. Comparatively, Moeini et al. reported that 71.46% of participants in Bobol (north of Iran) had used CAM in their lives [19]. The utilization of CAM in the USA increased from 28.9% in 1999 to 38.3% in 2007 [3].

The reported prevalence of complementary and alternative medicine (CAM) utilization (practice) was 48.5% in Australia and 49% in France [23, 24]. In Saudi Arabia, the utilization rate of CAM increased from 73% in 2008 to 84.6% in 2011 [25, 26], while among the Indian community in South Africa, this rate was 38.5% [22]. Furthermore, 31% of Finnish adults [27], 60% of Chinese [28], and 84% of Nigerians [29] have experienced using complementary medicine methods.

These comparisons suggest a lower prevalence of CAM use among the Iranian population in comparison to other countries. Despite previous studies concluding an increasing interest in CAM among the Iranian population [1, 11], our results indicate a relatively low percentage of CAM usage among Iranian adults.

We observed a statistically significant association between gender and age groups with PM use. Generally, females and individuals under 45 years old demonstrated the highest usage of PM. This aligns with the findings of several studies by Dehghan et al., Moeini et al., Von Conrady et al. and Al Akeel et al. In studies conducted in Kerman and Babol, southern and northern Iran, as well as in Australia and Saudi Arabia [19, 20, 30, 31], a statistically significant relationship between gender and CAM use was also revealed, similar to our study. In summary, our results suggest that Iranian adults possess low knowledge, relatively negative attitudes, and a low practice rate towards PM.

## Strengths and limitations

### Strengths

The present study boasted a large sample size and an exact sampling scheme, both of which heightened the generalizability of the findings.

### Limitations

1. The cross-sectional design of the study hindered the assessment of causality (e.g., between age and CAM use, and comparing these aspects with those of other countries).
2. The study relied on self-reported information, thus anticipating underreporting of certain questions.
3. Inherent limitations related to the recall of PM utilization experiences.

Finally, the availability of CAM services and providers is a critical consideration for analyzing the study’s results. Our study did not compare the results with the current map of CAM services in Iran due to the absence of such a map. Nonetheless, the existence of an educational environment where clinicians are trained and a highly educated staff is crucial for the availability of CAM therapies, which in turn is an undeniable factor when filling out the questionnaires.

## Conclusions

The current study shed light on a low level of knowledge, attitude, and practice toward PM among Iranian adults. The surveyed cases have low reliable knowledge (from medical staffs (2.9%) or books and other publications (15.3%)) and limited familiarity with PM, with about one third of the participants expressing disinterest (attitude) and over half of them having not utilized this form of medicine (practice).

Further studies are essential to investigate the reasons for the low utilization of PM and the prevalence of other types of complementary and alternative medicines among the population.

To enhance the overall population’s knowledge, attitude, and practice, specific strategies need to be formulated and implemented. Furthermore, it is crucial to conduct additional studies to probe into the efficacy of these intervention strategies.

## Data Availability

The datasets used and/or analyzed during the current study are available through the corresponding author upon reasonable request.

## Abbreviations

CAM: Complementary and alternative medicine
WHO: World Health Organization
PM: Persian Medicine
USA: United States of America
UK: United Kingdom
